# Self-Administered Botulinum Injection Causing Rare Masquerade of Systemic Botulism

**DOI:** 10.7759/cureus.64976

**Published:** 2024-07-20

**Authors:** Kai Wen Elvina Tay, Alvin Ong, Wei Ling Tay, Sze Joo Juan, Karen Chua

**Affiliations:** 1 Post-Acute and Continuing Care, Jurong Community Hospital, Singapore, SGP; 2 Emergency Medicine, Ng Teng Fong General Hospital, Singapore, SGP; 3 Rehabilitation Medicine, Tan Tock Seng Hospital, Singapore, SGP

**Keywords:** physical medicine and rehabilitation, muscle spasticity, adult botulism, botulism, botox injections, botox

## Abstract

This report describes the case of a patient who presented to the Emergency Department (ED) with a one-week history of difficulty in breathing, generalized weakness, dysphagia, and difficulty in walking. She had self-administered 100 units of onabotulinumtoxin A (BoNT-A) by injection into her face two weeks prior for cosmetic purposes. This case study highlights the rare but potential complication of systemic botulism.

## Introduction

Onabotulinumtoxin A (BoNT-A) injections are used commonly by Physical Medicine and Rehabilitation physicians in the treatment of post-stroke spasticity and in spasticity treatment for cerebral palsy in children [1,2]. BoNT-A injections are administered by dermatologists for the treatment of palmar and axillary hyperhidrosis and for cosmetic purposes [3]. It is also used to treat non-motor and motor symptoms of Parkinson's disease such as limb dystonia and sialorrhea [4]. Systemic botulism is a rare complication post botulinum toxin injection [5]. Understanding the clinical presentation, diagnostic approach, and treatment options is important for the effective management of systemic botulism. The main purpose of this case report is to raise awareness among physicians regarding the treatment and management of this rare complication, as well as diluent volumes, dosages and injection methods to reduce the risks of systemic botulism.

## Case presentation

A 46-year-old female with no significant past medical history presented to the Emergency Department (ED) with a one-week history of difficulty in breathing, generalized weakness, dysphagia, and difficulty in walking. She had no gastrointestinal symptoms, fever, or recent symptoms of illness such as acute respiratory infections. On further history taking, she reported having self-administered 100 units of BoNT-A via injection into her face two weeks prior for cosmetic purposes. She had purchased the BoNT-A from an overseas online shop and learned how to self-administer it due to her experience as a clinic assistant in an aesthetic medical clinic several years prior.

On clinical assessment, she was afebrile with normal vital signs. Neurological system examination revealed bilateral fixed ptosis, neck flexion weakness, and dysarthria, while manual muscle testing (MMT) of bilateral upper limb and lower limbs was 4/5 with normal sensorium.

**Figure 1:**
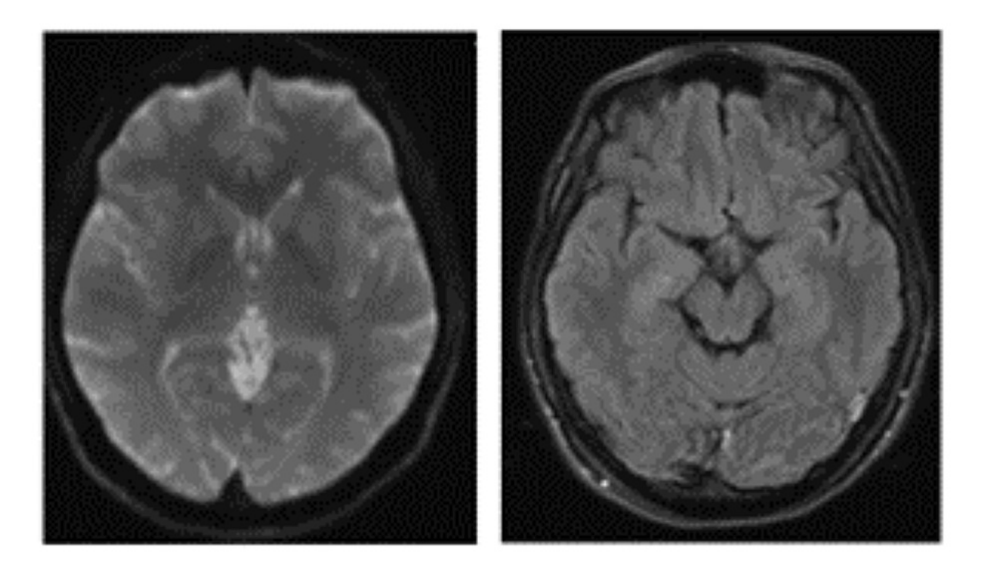
Magnetic resonance imaging of the brain showing T2 FLAIR and diffusion-weighted imaging sequences with no acute intracranial hemorrhage, acute infarct, or intracranial space-occupying lesion. There is no pathological intracranial contrast enhancement. FLAIR: fluid-attenuated inversion recovery

Laboratory investigations revealed normal creatine kinase and aldolase levels, negative acetylcholine receptor antibodies, normal thyroid panel, full blood count, renal function, and electrolyte levels (Table 1). Magnetic resonance imaging (MRI) brain had no significant abnormal findings (Figure 1). 

**Table 1:** Laboratory results of patient

Parameter	Values	Units	Reference values
White blood cells	4.85	X10^9^/L	4.30-10.40
Red blood cells	4.13	X10^9^/L	4.03-5.19
Haemoglobin	12.1	g/dl	11.5-14.9
Platelets	277	X10^9^/L	150-410
Absolute neutrophils	2.71	X10^9^/L	1.90-6.53
Absolute lymphocytes	1.62	X10^9^/L	1.21-3.56
Absolute eosinophils	0.08	X10^9^/L	0.05-0.50
Sodium	138	mmol/L	135-145
Potassium	4.1	mmol/L	3.5-5.2
Creatinine	79	umol/L	45-90
Urea	3.3	mmol/L	2.5-6.7
Chloride	107	mmol/L	95-110
Bicarbonate	24	mmol/L	22-32
Calcium	2.24	mmol/L	2.10-2.60
Phosphate	1.19	mmol/L	0.75-1.50
Magnesium	0.82	mmol/L	0.70-1.10
Albumin	38	g/L	35-52
Total bilirubin	8.6	umol/L	1.0-20.0
Alanine transaminase	20	U/L	6-35
Aspartate transaminase	26	U/L	6-30
Alkaline phosphatase	47	U/L	30-110
Creatine kinase	82	U/L	30-150
Aldolase	3.1	U/L	2.5-10.0
Free thyroxine	12.5	pmol/L	9.0-19.1
Thyroid-stimulating hormone	0.57	mU/L	0.35-4.94
High-sensitivity troponin I (hs-TNI)	<3.2	ng/L	<=26.2
Acetylcholine receptor antibodies	<0.07	nmol/L	<0.25 nmol/L
Drug toxicology screen	Acidic and Neutral Drugs: not detected; Benzodiazepines/Hypnotics and opioids: not detected; Other basic drugs panel not detected		

In the ED, she was referred to the intensive care unit team for concerns about the risks of progression to respiratory failure requiring ventilatory support. The patient was closely monitored in the general ward and remained stable. She did not require respiratory support. She was diagnosed with systemic botulism and was started on pyridostigmine. As the patient progressed well with no further significant clinical deterioration, she was not given the equine heptavalent antitoxin. In view of residual functional loss and requirement for assistance in basic activities of daily living (ADL) on a background of normal independent premorbid function, she was subsequently transferred to a community hospital after a nine-day stay in the acute general ward for further rehabilitation.

At the community hospital, she had proximal weakness over her bilateral upper limb and right lower limb with an MMT score of 4/5 but had normal reflexes and sensory examination. Bilateral ptosis was still present but neck flexion strength had improved. She had reduced effort tolerance and required minimal assistance in ambulation and contact guard assistance for her basic ADLs. She also had mild oropharyngeal dysphagia requiring a modified diet.

**Figure 2:**
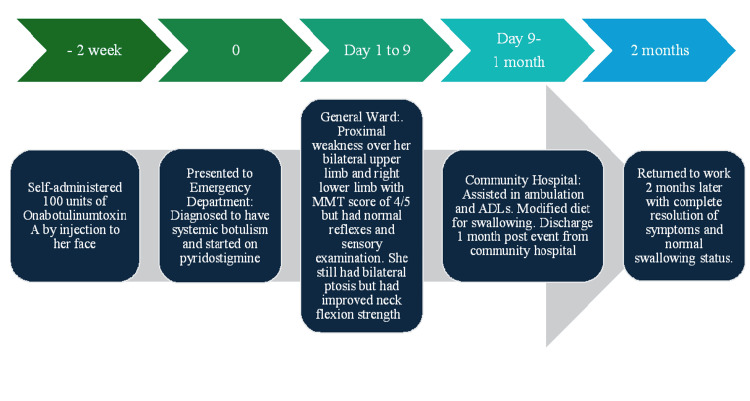
Timeline chart showing symptoms progression, treatment interventions, and recovery over time. MMT: manual muscle testing; ADL: activities of daily living

Having achieved marked functional improvement, she was discharged from the community hospital one month after her presentation and was independent in ambulation and basic ADLs but still required a modified diet. During the subsequent outpatient review, she reported having returned to work two months later with complete resolution of symptoms and a normal swallowing status. Figure 2 summarizes the patient's two-month recovery journey. 

## Discussion

BoNT-A is commonly used for cosmetic as well as therapeutic procedures such as the treatment of spasticity. It acts on the synaptosomal-associated protein of 25kDA (SNAP-25) toxin, thereby inhibiting acetylcholine release at the neuromuscular junction of striated muscles. Systemic botulism is rarely reported. One study suggested systemic spread occurred at a rate of 3.6 per 100 injection episodes [5]. The mechanisms of systemic botulism have been described as via hematogenous spread or neuroaxonal retrograde transport through the motor neurons of the spine [6].

As seen in this case, the clinical presentation of systemic botulism includes dizziness, difficulty breathing, and muscle weakness [7] and can manifest as symmetrical, descending flaccid paralysis [8]. Patients with systemic botulism can manifest with bulbar palsies, presenting with dysphonia, dysphagia, and dysarthria [9]. The most life-threatening complication of botulism is respiratory muscle weakness leading to respiratory failure [9]. Symptoms onset can occur days to weeks after injection depending on volume and dosage [7]. A study reported electromyography changes in muscles 3-13 days after BoNT-A injection [6].

Systemic botulism is a clinical diagnosis. Studies have reported that basic imaging and laboratory tests are often normal [7]. Cerebral spinal fluids can show elevated protein count but are often nondiagnostic [9]. Neurophysiological tests might reveal a reduction in compound muscle action potential (CMAP) amplitude and at least 20% facilitation of CMAP amplitude after tetanic stimulation [10]. These can be supportive but electrophysiological findings may not be present early in the disease [10].

The mainstay in the management of systemic botulism is supportive treatment. Mechanical ventilation and supportive care in an intensive care setting might be required while awaiting the recovery of respiratory muscles [11]. Early antitoxin administration, an equine heptavalent antitoxin, can prevent further disease progression but does not reverse symptoms. A case report suggested that early administration within 24 hours is best for foodborne and wound botulism. However, evidence of iatrogenic cases of botulism is limited [6]. It has been suggested that early administration of antitoxin might reduce respiratory complications [11]. A case series description of four iatrogenic botulism cases recorded early recovery of symptoms for three patients who received antitoxin early compared to a patient who had received it late [11].

Pyridostigmine is a reversible acetylcholinesterase inhibitor that prevents acetylcholine breakdown in the neuromuscular junction. It is commonly used in the treatment of myasthenia gravis. A study has suggested the use of pyridostigmine in cases of systemic botulism [12]. In this case, the patient was treated with pyridostigmine and reported an improvement in neck flexion strength after one week.

Some factors associated with systemic botulism in medical procedures such as spasticity treatment for post-stroke patients and cosmetic procedures include volume of dilution of BoNT-A, multiple point injection compared to a single bolus injection, guided rather than blind injection to technically avoid vascular access and to ensure accurate intramuscular placement of BoNT-A, and avoidance with concomitant use of certain medications such as aminoglycosides and those with known myasthenia gravis or neuromuscular junction disorders. It has been recommended a maximum dilution volume of 1 ml per injection site and a maximum dose per cycle of 400U for BoNT-A to reduce risks of systemic botulism at higher dilution volumes [13-15].

## Conclusions

This report highlights a rare case of severe systemic botulism from self-administered illicit BoNT-A with a full recovery. With regards to systemic botulism from medical injections, these are fortunately rare and preventable. Physicians performing medical procedures such as spasticity treatment using BoNT should be aware of proper prescribing and monitoring techniques to reduce the risks of complications of systemic botulism.
